# From the economics of TAVI and the pathophysiology of heart and vessel disease to metabolic disease in Africa and the developing world

**Published:** 2014-02

**Authors:** Paul A Brink

**Affiliations:** Department of Internal Medicine, Faculty of Health Sciences, University of Stellenbosch and Tygerberg Hospital, Tygerberg

The editor and staff of the journal welcome you, our readers, back in 2014. We trust that you enjoy the spread before your eyes.

Hailing from Angola, Magalhães *et al.* (page 27) looked at the prevalence of the metabolic syndrome in employees of a university, and emphasise the importance of waist circumference (WC) cut-off values that are appropriate for a specific ethnic group. The investigators used the clinical chemistry data obtained to derive WC values for this group. One should keep in mind that in Africa over the longer term, what we need is epidemiological studies to determine the extent to which the metabolic syndrome, in whatever way we define it, predicts cardiovascular events such as myocardial infarction, stroke and other events.

In one of two South African studies, health economics feature prominently. Mabin and Candolfi (page 21) compare costing of the relatively new intervention, transcatheter aortic valve implantation (TAVI), with conventional surgical aortic valve replacement (cAVR). Data were derived from these interventions in a private hospital group, a group where TAVI has been pioneered in South Africa, albeit with a major input from clinicians from university-associated public hospitals (Weich *et al.*^1^, Weich *et al.*^2^).

The second article (Freercks *et al.*, page 4) estimated central aortic systolic pressure (CASP) using a peripheral wrist-watchlike device BPro (HealthStats, Singapore). They found that CASP did not correlate with the degree of vascular calcification (VC), which is a risk factor for mortality in dialysis patients.

Authors from Iran and the Netherlands (Sattarzad *et al.*, page 34) tackled one of the holy grails in cardiac medicine using tissue Doppler, namely, an often-asked question: Is it the heart, the valves or the lungs that are responsible for a patient’s symptoms? In this instance they developed a Doppler-derived prediction model of left ventricular end-diastolic pressure (LVEDP) in the presence of known mitral valve stenosis.

Two articles emanate from Turkey. Gazi and fellow workers (page 9) examined endothelial function and dysfunction, and the association with polymorphisms in genes involved in endothelial function in patients with coronary slow flow (CSF). CSF is an angiographic phenomenon of delayed passage of contrast along the coronary arteries in the absence of stenosis in the epicardial arteries. As a proxy for endothelial function they used flowmediated dilatation (FMD) of the brachial artery.

Altun *et al.* (page 15) worked from the premise that an increased risk of atrial fibrillation may be related to cardiac changes during pregnancy. They showed that P-wave dispersion and certain tissue Doppler-derived parameters of electromechanical coupling were different from those in the controls.

The issue is complemented by online publication of three case reports. All in all a nice little feast with which to start the year.
